# Editorial: Insights in plant-pathogen interactions: 2023

**DOI:** 10.3389/fpls.2025.1547600

**Published:** 2025-01-28

**Authors:** Choong-Min Ryu, Brigitte Mauch-Mani

**Affiliations:** ^1^ Infectious Disease Research Center, Korea Research Institute of Bioscience and Biotechnology (KRIBB), Daejeon, Republic of Korea; ^2^ Department of Biosystems and Bioengineering, KRIBB School, University of Science and Technology, Daejeon, Republic of Korea; ^3^ Department of Biology, Université de Neuchâtel, Neuchâtel, Switzerland

**Keywords:** plant pathology, pathogen, defense signaling, resistance, plant immunity

## Introduction

We are now well into the second decade of the 21st Century, and, especially in recent years, the achievements made by scientists have been exceptional, leading to major advancements in the fast-growing field of plant science. To reflect this development, a Research Topics highlighting the latest advancements in this research field, with articles by members of the accomplished editorial board of Frontiers in Plant Science, section Plant-Pathogen Interactions, was initiated. This Research Topic of particular relevance was led by the two Specialty Chief Editors of the Plant-Pathogen Interactions section. It focusses on new insights, novel developments, current challenges, latest discoveries, recent advances, and future perspectives in the field of Plant-Pathogen Interactions. This Research Topic will inspire, inform, and provide direction and guidance to researchers in the field.

## What are the causes of plant diseases?

In the field of plant-pathogen interactions, the study on virulence and pathogenicity of microbial pathogens has always been of special interest. Not surprisingly, in this Research Topic too, many of the submitted papers focused on this area addressing diseases caused by oomycetes, fungi, bacteria and viruses.

Deng et al., reported on a new virulence factor, the cell wall degrading enzyme pectinesterase (PE) secreted by *Phytophthora infestans*, the well-known Oomycete that caused the Irish famine I the mid 19^th^ century. PE activity and gene expression were shown to be positively correlated to the increased pathogenicity of *P. infestans* on potato.

Several reports focused on how fungi cause diseases on their hosts. The grapevine trunk disease, Botryosphaeria dieback, is the cause of large economic losses in France for lack of effective treatments. Moret et al. demonstrated that members of the *Botrysphaeriaceae* family present in the vascular system in the basal internodes of stems in grapevine impact on the physiology and development of xylem and phloem of basal stem internodes resulting in an obstruction of the sieve plates by callose and symptoms such as leaf drop and premature plant death.

*Colletotrichum viniferum*, the causal pathogen of grape ripe rot and leaf spot, also has an important impact on grape production. Dou et al. evaluated the differences between highly sensitive, moderately resistant and resistant grapevine cultivars at the cytological upon infection with this pathogen and analyzed changes in the transcriptome of susceptible and resistant grapevine cultivars following *C. viniferum* challenge. They identified 236 differentially expressed *C. viniferum* genes encoding among others effector proteins. On the plant side differentially expressed grape genes showed differences in hormone signaling, phytoalexin production or disease-related genes.

The effector proteins secreted during another foliar disease, Brown rot, caused by necrotrophic plant pathogens *Monilinia* spp. in many stone fruits were evaluated to elicit cell-death phenotype on the non-host plants tobacco and tomato and the host plant *Prunus* spp. (Lopez et al.). This proceeding allows the screening of *Prunus* germplasm to identify susceptibility to brown rot.

Besides above-ground pathogens, the soil-borne pathogens are a further challenge affecting crop productivity. One of the serious soil-borne pathogens, *Fusarium* species makes devastating damages to the roots and stalks of maize in the field. Xiong et al. investigated which plant organs such as ears, stalks, and roots could be the place where the highest symptom-genotype correlation occurred. Out of 43 *Fusarium* spp. isolates, *Fusarium verticillioides* showed a strong correlation with stalk and root rot, but not with ear rot, indicating the main battlefield for the coevolution between maize and *F. verticillioides* is in the stalks and roots of maize.

In the interaction between pathogenic bacteria and host plants, studies on effector proteins to understand the gene-for-gene theory and immune response and modulation made significant progress in last three decades. Here, the first identified bacterial effector *AvrRps4* and its family was reviewed by Horton and Gassmann as the 30^th^ anniversary of the discovery and cloning of *avrRps4* approaches.

Interesting data on the bacterial effector gene *eop1* from *Erwinia amylovora* and the FB_Mar12 resistance locus of apple point to a putative relationship between the two. This could be exploited to generate fire blight resistant apple varieties (Emeriewen et al.).

Because of global warming and globalization, new and re-emerging virus diseases occur due to the expanding habitat of insect vectors. Wang et al. demonstrated that the coat protein of Citrus yellow vein clearing virus interacts with the ascorbate peroxidase 1 (ClAPX1) from lemon plants resulting in an increased virus accumulation and a decreased expression of most genes involved in jasmonic acid signaling.

A study on the virulence factor of Mungbean yellow mosaic virus in mung bean (*Vigna radiata*) validated the infectivity of a series of infectious clones (Balasubramaniam et al.). In the future, this information can be used to identify cultivars susceptible and resistant to yellow mosaic disease.

## How do plants interact with pathogen and protect themselves?

In nature, plants need to recognize potential pathogens and act appropriately to pathogen attacks. Plant defense response and resistance mechanisms are an important topic in the plant-pathogen interaction field. Here, the critical review of a novel signaling molecule, the CLAVATA3/EMBRYO SURROUNDING REGION-RELATED (CLE) peptide derived from the *CLE* gene in many plant species was shown to play a role during nodulation, immunity, and symbiosis with arbuscular mycorrhizal fungi. This sheds light on the role of regulatory networks on trade-off between plant defense and growth (Nakagami et al.).

The analysis of PR proteins in the cocoa tree apoplastome during infection by *Moniliophthora perniciosa* shows the defense dynamics occurring during this interaction. This could help designing novel control strategies for witches’ broom disease (De Oliveira et al.).

Leucine-rich repeat receptor-like kinases are also prominent actors in plant defense. Yan et al. showed that the *Sesamum indicum* SiLRR-RLKs play a vital role in biotic stress and contribute to the sesame plant resistance to *Macrophomina phaseolina*.

## How can plant diseases be managed?

The major objective to study plant-microbe interaction and plant pathology is to protect crops against pathogens. In this Research Topic, novel biological and synthetic materials are shown to control foliar and soil-borne pathogens. Intriguingly, amphibian skin bacteria application inhibits gray mold caused by *Botrytis cinerea* on tomato and post-harvest blueberries as well as the model plant *Arabidopsis thaliana* (Romero-Conttreras et al.) and root-associated bacteria (rhizobacteria) were shown to activate soybean chitinases and defense mechanism against *Fusarium oxypsorum* (Chen et al.).

Chitosan, a biopolymer extracted from crustacean exoskeletons has been used to control downy and powdery mildews in grapevine. The authors show that chitosans with a low degree of polymerization are effective inducers of defense in grapevine and also exhibit strong pesticidal effects against *Botrytis cinerea and Plasmopara viticola* (Brule et al.).

An ecological point of view can give hints on how to manage plant diseases. Investigation on the microbial transition and network studies between phyllosphere and rhizosphere suggest that interactions among bacteria of the genera *Caulobacter* and *Bosea* could help to manage wildfire disease in hydroponic systems (Liu et al.).

Novel data on salicylic acid-dependent plant signaling in age-related resistance of *Arabidopsis* suggest that salicylic acid accumulation and response are transiently increased during leaf maturation. A similar pattern was observed in cotton plants (Hu et al.).

High Ca2+ influx was shown to regulate ferroptotic cell death in rice. It triggered iron-dependent ROS accumulation, lipid peroxidation, and subsequent hypersensitive response cell death (Wang et al.).

## Outlook

The Research Topic solicited brief, forward-looking contributions from the editorial board members that describe the state of the art, outlining recent developments and major accomplishments that have been achieved and that need to occur to move the field forward for the first time in our section. The goal of this special edition Research Topic was to shed light on the progress made in the past decade in the Plant-Pathogen Interactions field, and on its future challenges to provide a thorough overview of the field. The current Research Topic was successful and timely provides the global research field trends in the plant -pathogen interaction area.

